# Plasma levels of VIP are not elevated during PACAP- and VIP-induced cluster headache attacks: an exploratory study

**DOI:** 10.3389/fneur.2023.1135246

**Published:** 2023-04-18

**Authors:** Christina Deligianni, Lanfranco Pellesi, Basit Ali Chaudhry, Anne Luise Haulund Vollesen, Agneta Henriette Snoer, Jens Hannibal, Rigmor Højland Jensen, Messoud Ashina

**Affiliations:** ^1^Danish Headache Center, Department of Neurology, Rigshospitalet Glostrup, Faculty of Health and Medical Sciences, University of Copenhagen, Copenhagen, Denmark; ^2^Department of Clinical Biochemistry, Bispebjerg Frederiksberg Hospital, Faculty of Health Sciences, University of Copenhagen, Copenhagen, Denmark

**Keywords:** cluster headache, PACAP38, VIP, headache, migraine, pain, parasympathetic system

## Abstract

**Background:**

Pituitary adenylate cyclase-activating peptide (PACAP) and vasoactive intestinal peptide (VIP) provoked cluster headache attacks in individuals with episodic cluster headache during their active phase and individuals with chronic cluster headache. In this study, we investigated whether infusions of PACAP and VIP caused alterations in plasma levels of VIP and their potential contribution to induced cluster headache attacks.

**Methods:**

Participants received either PACAP or VIP infusion for 20 min on 2 separate days with an interval of at least 7 days in between. Blood collection was performed at T_0_, T_20_, T_30_, and T_90_. Plasma levels of VIP were measured using a validated radioimmunoassay method.

**Results:**

Blood samples were collected from participants with episodic cluster headache in the active phase (eCHA, *n* = 14), remission (eCHR, *n* = 15), and from participants with chronic cluster headache (cCH, *n* = 15). Baseline levels of VIP did not differ among the three groups (*p* = 0.1161). During PACAP infusion, mixed-effects analysis revealed a significant increase in plasma levels of VIP in eCHA (*p* = 0.0300) and eCHR (*p* = 0.0058) but not in cCH (*p* = 0.2930). We found no difference in the increase of plasma VIP levels between patients who developed PACAP38- or VIP-induced attacks.

**Conclusion:**

Cluster headache attacks induced by PACAP38 or VIP infusion are not associated with changes in plasma levels of VIP. Further studies are needed to investigate the role of VIP and the parasympathetic system in cluster headache.

**Clinical trial registration:**

The parent study is registered at ClinicalTrials.gov (NCT03814226).

## Introduction

Cluster headache is typically characterized by cluster periods of recurrent attacks of unilateral head pain associated with prominent unilateral autonomic symptoms (1). Pathophysiological mechanisms of cluster headache are complex and include the activation of the trigeminovascular system (2). Recent studies suggested an important role for pituitary adenylate cyclase-activating polypeptide (PACAP) and vasoactive intestinal polypeptide (VIP) in the pathogenesis of cluster headache (3). Both peptides are expressed in the parasympathetic ganglia and are released upon activation of the trigeminovascular system (4), whereas PACAP is also expressed in trigeminal neurons (5, 6). The role of VIP in cluster headache is not fully clarified. While one study reported elevated plasma VIP during spontaneous attacks (7), another study reported no increase in VIP levels during cluster headache attacks induced by calcitonin gene-related peptide (CGRP) (8). Recently, we demonstrated that intravenous infusion of PACAP38- and VIP-induced cluster headache attacks during cluster periods (9). Whether plasma levels of VIP are elevated during PACAP- and/or VIP-induced cluster headache attacks is unknown. In this exploratory study, we investigated potential plasma changes in VIP during PACAP- and VIP-induced cluster headache attacks.

## Materials and methods

Participants were recruited from the Danish Headache Center (Rigshospitalet Glostrup) between December 2017 and August 2019. Inclusion criteria were as follows: age between 18 and 65 years old and diagnosis of episodic or chronic cluster headache according to the International Classification of Headache Disorders third edition (beta version) (1). Participants with episodic cluster headache in the active phase were defined by typical cluster headache attacks within the last 30 days. People in remission were defined by no cluster headache attacks for at least 30 days. Participants with chronic patients were defined by the presence of attack-free periods lasting not more than 1 month in the last 12 months. Exclusion criteria were as follows: history of other primary headaches (except for episodic tension-type headache <5 days/month or migraine if >12 months from the last attack), any significant somatic or psychiatric disease, drug abuse, pregnancy, or breastfeeding. Women with childbearing potential were tested with a urine pregnancy test before both provocation days. The intake of any preventive treatment was allowed without changing the dosing, except from steroids in the last 30 days.

The study is part of a previously conducted parent study (3) approved by the Ethical Committee of the Capital Region of Denmark (H-17011659) and the Danish Data Agency, in accordance with the Declaration of Helsinki. The study was registered on www.clinicaltrials.gov (NCT03814226). All participants signed consent forms after receiving oral and written information about the study.

### Study design

The study was conducted as a randomized, double-blind, cross-over study. We included patients with episodic cluster headache in the active phase (eCHA), episodic cluster headache patients in remission (eCHR), and chronic cluster headache patients (cCH). Before the experiment, eCHA and cCH patients were attack-free for more than 4 h. All patients were randomized and allocated to receive a continuous infusion (Braun Perfusor, Melsungen, Germany) of either PACAP38 (10 pmol/kg/min) or VIP (8 pmol/kg/min) over 20 min on 2 experimental days, with an interval of at least 7 days in between. PACAP38 and VIP were prepared in identical vials and randomized by the Regional Central Pharmacy. During the study, the randomization code remained in the hospital and was unavailable to blinded medical investigators until the study was completed and data were analyzed.

### Study procedures

Physical and neurological examinations, together with an ECG assessment, were conducted before the first experimental day. Experiments were conducted with participants staying in the supine position, with two venous catheters (Venflon) in the right and left antecubital vein for infusion and blood sampling purposes. Baseline measurements related to headache intensity, accompanying symptoms, and vital signs (blood pressure and heart rate) were collected after the resting period of 15 min. Blood sampling was performed at fixed time points: at the baseline (T_0_), at the end of the infusion (T_20_), 10 min after the end of the infusion (T_30_), and at T_90_. If the participants developed a cluster headache attack during the observational period, an extra blood sampling was performed at the onset of the attack (T_a_).

### Blood sampling and processing

At fixed time points, 5 ml of blood was collected and discarded. Then, blood sampling was performed with a 20 ml syringe and the intravenous catheter was flushed with 10 ml of normal saline.

The blood was then moved into pre-cooled lithium heparin tubes containing aprotinin (Trasylol^®^). All tubes were gently inverted 5–6 times and stored at 5°C for 20 min until centrifugation (1,600 g for 10 min). Then, plasma was moved to cryotubes (Thermo Fisher Scientific, Jiangsu, China) and stored at −80°C until analysis. At the moment of the analysis, laboratory technicians were blinded to the randomization lists.

### Radioimmunoassay

Plasma VIP was measured with a validated radioimmunoassay (Diasource, Louvain-la-Neuve, Belgium), as previously described (10). When plasma levels of VIP were below the limit of detection (3.8 pmol/l), we replaced the readings with a fixed value (3.8 / 2 = 1.9 pmol/l) as previously suggested (11).

### Statistical analysis

All absolute values are presented as mean ± standard deviation (SD) or median and interquartile range (IQR). The exploratory endpoints were as follows: (a) any change of plasma VIP as a result of VIP and PACAP infusions in eCHA, eCHR, and cCH patients; (b) any change of plasma VIP during experimentally induced attacks; and (c) any difference of plasma VIP at the baseline among cluster headache patients plus a historical group of interictal episodic migraine patients (10). Regarding (a), we used a mixed-effect model to evaluate any change in plasma VIP during PACAP and VIP days. The Greenhouse–Geisser correction was applied to adjust the lack of sphericity. Tukey's *post-hoc* test was used to correct for multiple comparisons. Therefore, adjusted *p*-values are reported. Regarding (b), we calculated the sum of the differences between plasma VIP at the baseline and the following time points after PACAP38 and VIP infusions. In this way, we obtained a summary score of the changes in plasma VIP during each experimental day. Then, we compared the summary scores between individuals who reported a cluster headache attack and those who did not by using the Kolmogorov–Smirnov test. Regarding (c), we used the Brown–Forsythe ANOVA test to compare the plasma levels of VIP at the baseline among the four groups. We used the R software (version 4.0.2) and the GraphPad Prism software (version 9.0.2) for statistical analysis and graphs. All *p*-values were considered significant if *p* < 0.05.

## Results

**Figure 1:**
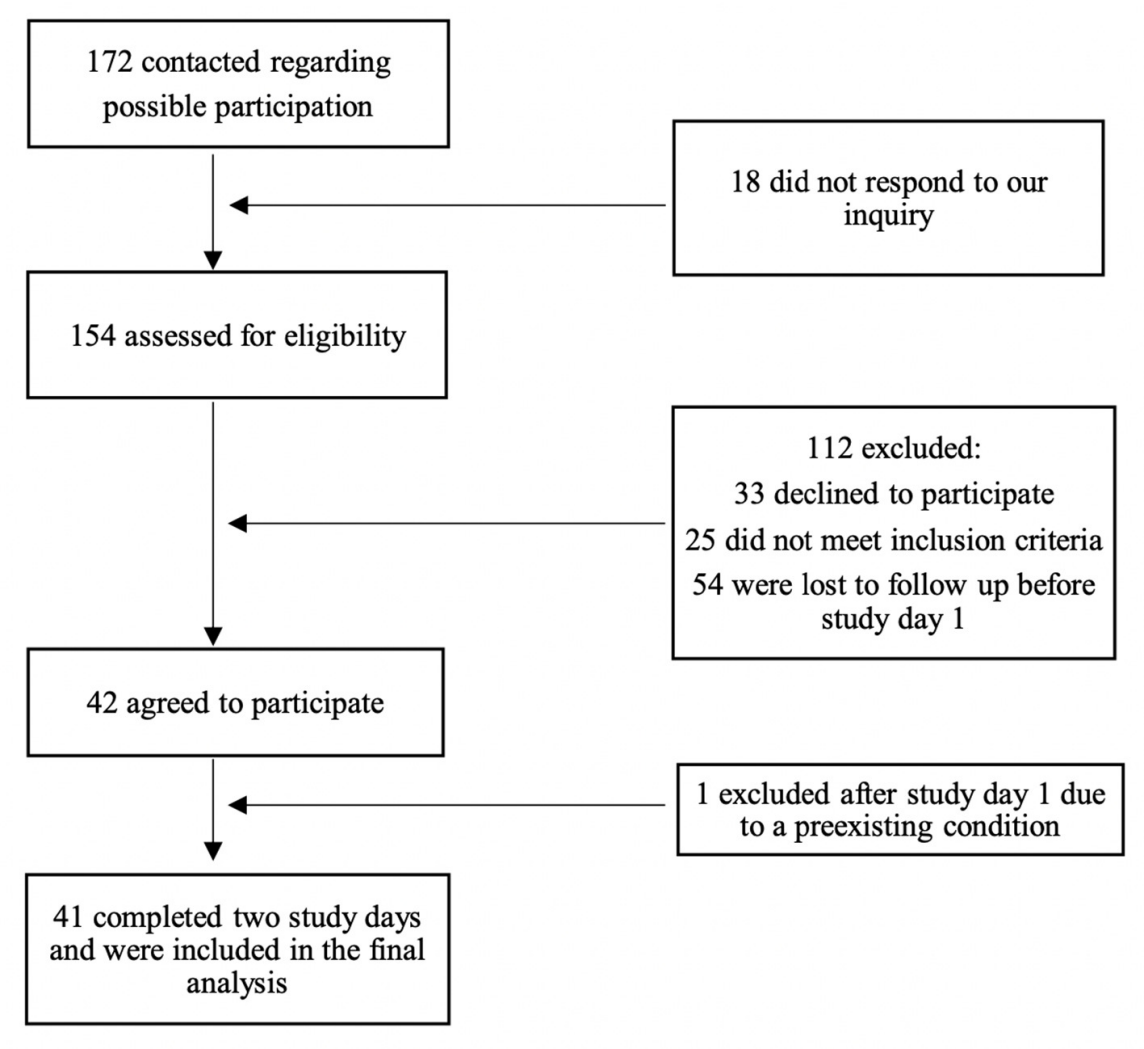
Flowchart of the study.

We enrolled 14 eCHA participants, 15 eCHR participants, and 15 cCH participants (Figure 1). The demographic and clinical characteristics of all participants are presented in Table 1. In total, three individuals with eCH participated in both the active and remission phases and were included in both eCHA and eCHR groups, respectively.

**Table 1:** Clinical characteristics of enrolled patients.

	**Sex (F/M)**	**Age (mean ±SD)**	**BMI (mean ±SD)**	**Years with CH (mean ±SD)**	**Preventive treatment (n)**
Episodic CH in active phase (*n* = 14)	4/10	35.4 ± 10.0	24.5 ± 3.5	15.5 ± 10.1	9^*^
Episodic CH in remission (*n* = 15)	1/14	36.7 ± 9.9	24.9 ± 3.2	14.9 ± 8.7	2^**^
Chronic CH (*n* = 15)	2/13	43.5 ± 13.5	23.6 ± 2.9	12.2 ± 9.5	9^***^

Age is expressed as years, body mass index (BMI) is expressed as kg/m^2^. SD, standard deviation; F/M, female/male; CH, cluster headache.

^*^Verapamil (between 100 and 800 mg), lithium, and gabapentin.

^**^Verapamil (between 120 and 160 mg).

^***^Verapamil (between 1,220 and 180 mg), lithium, gabapentin (between 300 and 2,700 mg), and melatonin.

A total of six out of 14 (43%) eCHA participants reported cluster headache after PACAP infusion, while five out of 14 (36%) eCHA participants reported cluster headache after VIP infusion. In the cCH group, seven out of 15 (47%) participants reported cluster headache after either PACAP or VIP infusion. None of the eCHR participants reported cluster headache during experimental days. All CH individuals reported that PACAP- and VIP-induced CH attacks were phenotypically similar to their usual spontaneous ones. Clinical characteristics of the provoked cluster headache attacks are shown in Table 2. The missing values of plasma VIP were four out of 352 planned samples (1.1%). The mean plasma concentration of VIP during PACAP and VIP days among all groups is reported in Table 3.

**Table 2:** Clinical characteristics of PACAP- and VIP-induced cluster headache.

		**Time to onset**	**Duration**	**Peak headache intensity**	**Acute therapy**	**Accompanying symptoms**
eCHA 01	PACAP38	40	10	4	Sumatriptan	Lac/inj/con/mio
eCHA 02	PACAP38	40	20	4	Sumatriptan	Con/ede
eCHA 03	VIP	60	40	1	No	Lac/con/pto
eCHA 07	PACAP38	10	60	9	Oxygen	Lac/con
eCHA 08	VIP	90	10	3	No	Lac
eCHA 09	PACAP38	10	10	6	Sumatriptan (2 doses)	Lac/con
eCHA 09	VIP	20	20	6	Sumatriptan (2 doses)	Lac/inj/con/ede/mio
eCHA 10	PACAP38	50	30	3	No	Lac/inj/con/swe
eCHA 10	VIP	70	30	2	No	Lac
eCHA 13	PACAP38	30	60	7	Sumatriptan (2 doses)	Rhi/con
eCHA 13	VIP	60	30	4	Sumatriptan	Con
cCH 01	PACAP38	50	20	1	No	Lac
cCH 02	PACAP38	70	30	2	No	Lac/rhi/con
cCH 02	VIP	50	50	3	Oxygen	Lac/con
cCH 03	PACAP38	40	60	7	Oxygen and sumatriptan	Lac/rhi/con
cCH 03	VIP	40	60	7	Sumatriptan	Pto
cCH 04	PACAP38	20	40	4	No	Rhi/ede
cCH 04	VIP	10	60	4	No	Ede
cCH 07	PACAP38	50	30	4	Sumatriptan	Rhi/pto/mio
cCH 07	VIP	30	70	1	No	Lac
cCH 08	PACAP38	70	30	3	No	Lac
cCH 08	VIP	10	90	5	Sumatriptan	Lac/pto
cCH 12	VIP	30	20	3	No	Mio
cCH 14	PACAP38	20	40	3	No	Lac/con
cCH 15	VIP	30	40	2	No	Lac/con

Time to onset and duration: minutes, starting with the experimental infusion. Peak headache intensity was measured by using a visual analog scale from 0 to 10. Sumatriptan, sumatriptan 6 mg sc; Con, nasal congestion; Ede, eyelid edema; Inj, conjunctival injection; Lac, lacrimation; Mio, miosis; Pto, ptosis; Rhi, rhinorrhea; Swe, forehead and facial sweating.

### Plasma VIP during PACAP days

**Figure 2:**
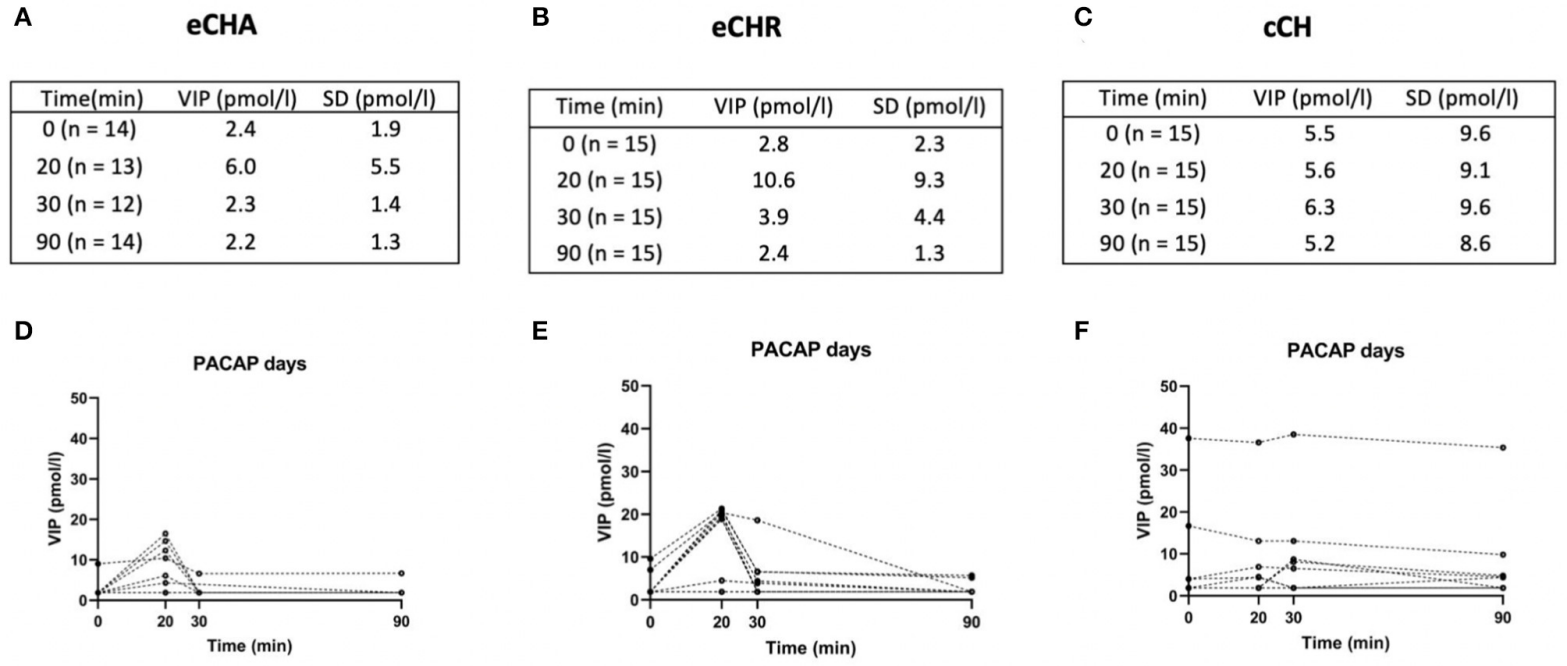
Plasma levels of VIP (pmol/l) during PACAP38 days. **(A–C)** Mean plasma levels of VIP among the three groups. **(D–F)** Individual values among the three groups.

**Figure 3:**
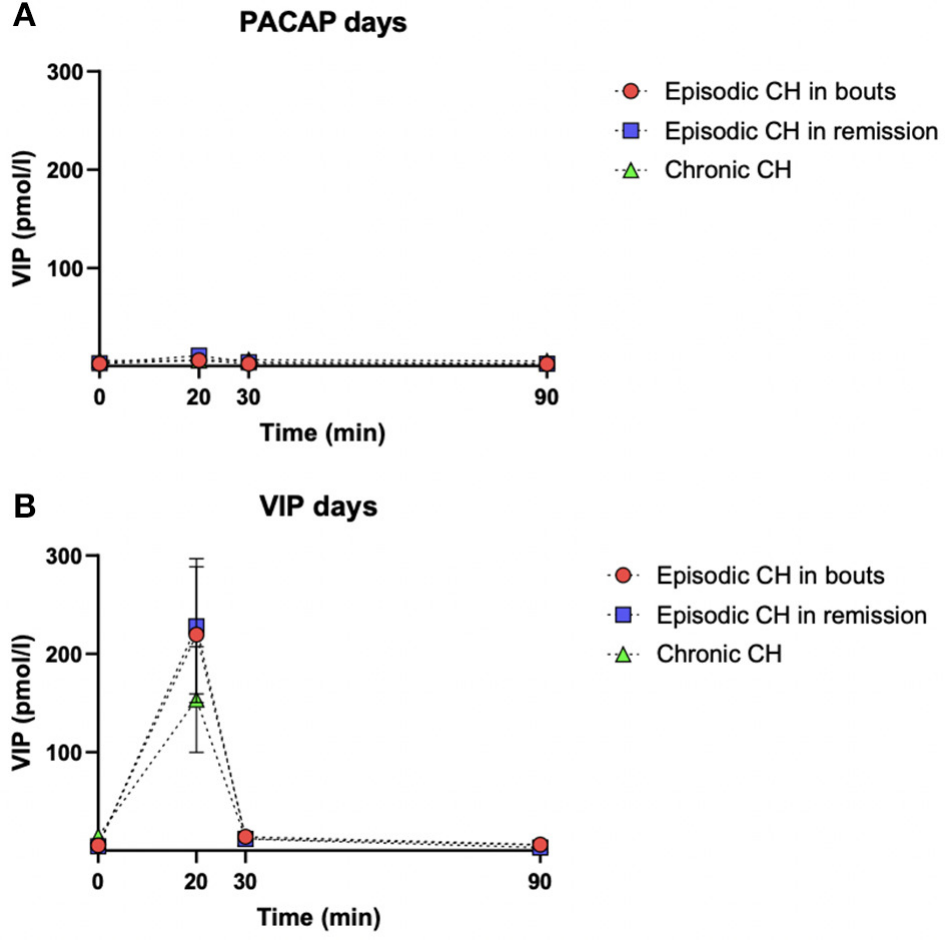
Mean plasma levels and standard errors of VIP (pmol/l) for each study group during PACAP38 **(A)** and VIP days **(B)**. The dashed black line with red dots represents eCHA patients, the dashed black line with blue squares represents eCHR patients, and the dashed black line with the green triangle represents cCH patients.

**Figure 4:**
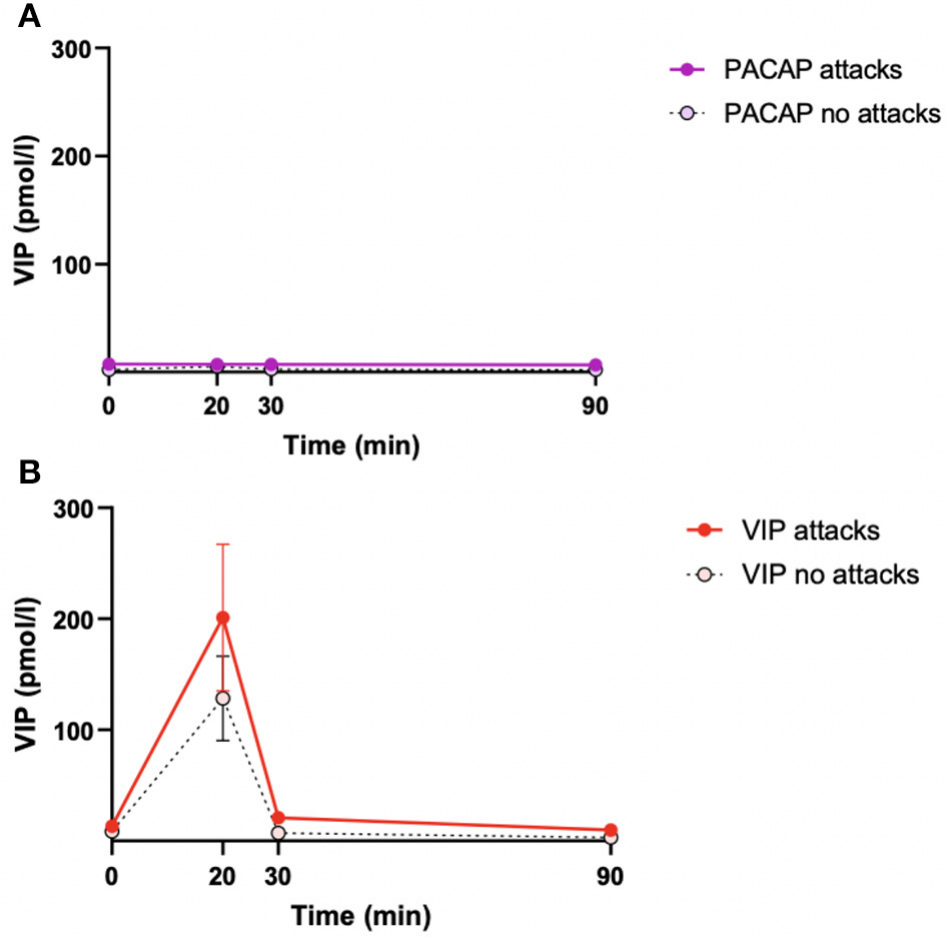
Mean plasma levels of VIP in patients reporting cluster headache and attack-free patients during PACAP days **(A)** and VIP days **(B)**. We included eCHA and cCH patients. The solid lines with red/purple dots represent the average of individuals who reported cluster headache. The dashed black lines with white dots represent the average of individuals who did not develop cluster headache. Error bars are the standard errors of the mean.

During PACAP38 infusions, plasma levels of VIP significantly changed in eCHA (*p* = 0.030) and eCHR (*p* = 0.006) participants but not in cCH (*p* = 0.293) participants (Figure 2). The peak plasma concentration of VIP was measured at T_20_ in both groups (eCHA = 6.0 ± 5.5 pmol/l, eCHR = 10.6 ± 9.3 pmol/l) but not in cCH group (cCH_20_ = 5.6 ± 9.1 pmol/l) (Figure 3A). Considering eCHA and cCH participants, we found no difference in plasma VIP between individuals who developed an attack (n = 11) compared with those who did not (*n* = 16) (*p* = 0.3547) (Figure 4A).

### Plasma VIP during VIP days

**Figure 5:**
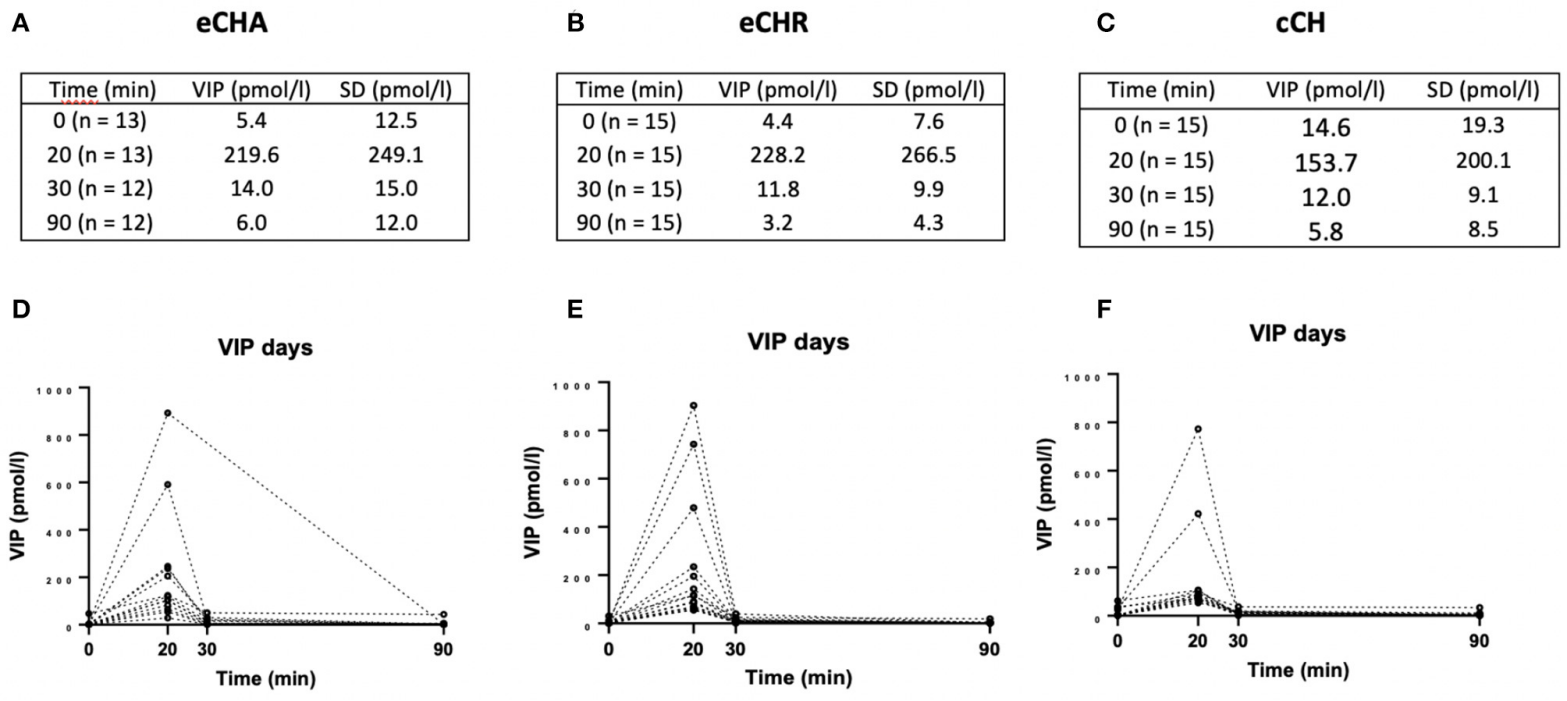
Plasma levels of VIP (pmol/l) during VIP days. **(A–C)** Mean plasma levels of VIP among the three groups. **(D–F)** Individual values among the three groups.

On VIP days, plasma levels of VIP significantly changed in the three groups (eCHA, *p* = 0.012; eCHR, *p* = 0.002; cCH, *p* = 0.018) (Figure 5). The peak plasma concentration of VIP was measured at T_20_ in all groups (eCHA = 219.6 ± 249.1 pmol/l, eCHR = 228.2 6 ± 266.5 pmol/l, cCH = 153.7 ± 200.1 pmol/l) (Figure 3B). Considering eCHA and cCH participants, we found no difference in plasma VIP between participants who developed an attack (*n* = 11) vs. those who did not (*n* = 14) (*p* = 0.317) (Figure 4B).

### Baseline levels of VIP

**Figure 6:**
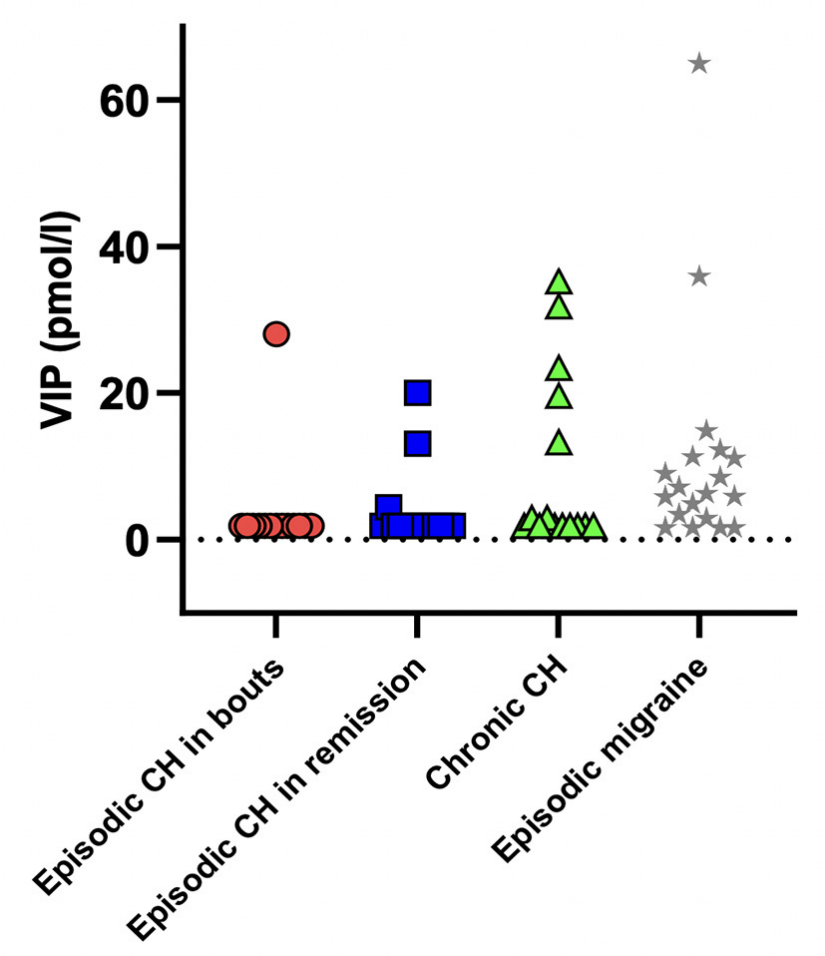
Individual plasma levels of VIP at the baseline. No significant difference was found among cluster headache patients and a historical group of interictal episodic migraine patients (*p* = 0.1161). Mean plasma levels ± standard deviation: eCHA = 3.764 ± 6.976; eCHR = 4.023 ± 5.291; cCH = 9.633 ± 12.010; EM = 11.080 ± 15.200. eCHA: episodic cluster headache in bouts, eCHR: episodic cluster headache in remission, cCH: chronic cluster headache patients, EM: episodic migraine patients.

We found no difference in plasma VIP between eCHA (*n* = 14) (3.764 ± 6.976 pmol/l, IQR 1.90–28.00), eCHR (*n* = 15) (4.023 ± 5.291 pmol/l, IQR 1.90–20.00), cCH (*n*= 15) (9.633 ± 12.01 pmol/l, IQR 1.90–35.25), and a historical group of people with migraine outside of attacks (*n* = 19) (11.08 ± 15.20, IQR 1.60–64.95) (*p* = 0.1161). Individual baseline values are displayed in Figure 6.

## Discussion

The present study revealed no changes in plasma levels of VIP during PACAP- and VIP-induced cluster headache attacks. Of interest, plasma levels of VIP were elevated in individuals with eCHA and eCHR but not in cCH following PACAP infusions. At baseline, plasma VIP did not differ between the three groups of cluster headache individuals and in comparison to a historical group of people with migraine (11).

### Plasma VIP during cluster headache attacks

During spontaneous attacks in eCH, increased plasma levels of VIP were reported when blood was collected from the external jugular vein ipsilateral to the attack (7). In the present study, we found no changes in plasma VIP during PACAP- and VIP-induced cluster headache. Similar findings were reported during CGRP-induced cluster headache attacks (8). Consistent with previous studies, we collected blood from the antecubital vein. In healthy volunteers, plasma VIP did not differ when collected in the external jugular, internal jugular, and antecubital vein (12).

### Plasma VIP during PACAP experimental days

During PACAP days, we found elevated plasma levels of VIP at T_20_ during the active phase and remission. Interestingly, after the 20-min infusion of PACAP38, plasma levels of VIP returned to baseline values. *In vitro*, PACAP induced an increased expression of VIP in human neuroblastoma cells, suggesting that PACAP released from nerve terminals could influence the function of VIPergic neurons in target tissues (13). Furthermore, PACAP infusion caused an increase in plasma levels of VIP in people with migraine (14). Interestingly, plasma VIP did not change in participants with cCH patients during PACAP infusions. These results suggest that plasma levels of VIP may fluctuate depending on disease activity, with different mechanisms contributing to the generation of an attack. Similarly, CGRP infusion resulted in an increase in plasma VIP regardless of disease activity (8). Nevertheless, the current study is exploratory, and such results should be interpreted with caution. The increase in plasma levels of VIP in eCHA and eCHR was only modest. In cCH patients, no alteration in plasma levels was detected. This discrepancy could be explained by a small sample size or possible differences in neuropeptide synthesis between patients and healthy volunteers.

### Study limitations

This study has some limitations. The design of the study was exploratory, with the sample size being estimated to detect clinically relevant differences between PACAP- and VIP-induced attacks. The implemented statistical tests are appropriate, but the chosen sample size might not be able to fully evaluate our assumptions. In addition, our assay might have cross-reacted with PACAP38, explaining the higher plasma levels of VIP during PACAP days. However, the assay has been validated against cross-reaction with PACAP, making this hypothesis less likely.

## Conclusion

We showed that plasma levels of VIP did not change during PACAP- and VIP-induced cluster headache attacks. Of interest, PACAP infusions elevated plasma levels of VIP in eCHA and eCHR patients but not in cCH patients. These findings generate further interest in clarifying the role of PACAP38 and VIP in cluster headache. It would also be interesting to investigate plasma levels of PACAP during provoked cluster headache.

## Data availability statement

The raw data supporting the conclusions of this article will be made available by the authors, without undue reservation.

## Ethics statement

The studies involving human participants were reviewed and approved by Ethical Committee of the Capital Region of Denmark (H-17011659) and the Danish Data Agency. The patients/participants provided their written informed consent to participate in this study.

## Author contributions

Study concept and design: MA, AH, AS, and RJ. Final approval of the completed manuscript: MA. Revising for intellectual content: MA, CD, LP, BC, AH, AS, RJ, and JH. Drafting of the manuscript: MA, CD, and LP. Analysis and interpretation of data: MA, LP, CD, and JH. Acquisition of data: MA, AH, AS, BC, and RJ. All authors contributed to the article and approved the submitted version.
